# Prevalence of “unclassified” HPV genotypes among women with abnormal cytology

**DOI:** 10.1186/s13027-018-0199-0

**Published:** 2018-07-24

**Authors:** Clorinda Annunziata, Giovanni Stellato, Stefano Greggi, Veronica Sanna, Maria Pia Curcio, Simona Losito, Gerardo Botti, Luigi Buonaguro, Franco Maria Buonaguro, Maria Lina Tornesello

**Affiliations:** 1 Molecular Biology and Viral Oncology Unit, Istituto Nazionale Tumori IRCCS “Fondazione G. Pascale”, via M Semmola, 80131 Naples, Italy; 2 Gynecology Oncology Unit, Istituto Nazionale Tumori IRCCS “Fondazione G. Pascale”, 80131 Naples, Italy; 3 Department of Pathology, Istituto Nazionale Tumori IRCCS “Fondazione G. Pascale”, 80131 Naples, Italy

**Keywords:** Human papillomavirus, Cervix carcinoma, Squamous intraepithelial neoplasia

## Abstract

**Background:**

High risk human papillomaviruses (HPVs) have been unequivocally recognised as the necessary cause of squamous intraepithelial lesions (SIL) and invasive carcinoma of the cervix. The distribution and the role of unclassified risk HPV genotypes in cervical neoplasia has not been fully elucidated.

**Methods:**

Liquid-based cytological samples were collected from 337 women referred for colposcopy following an abnormal cytological diagnosis. HPV DNA was detected by broad-spectrum PCR and genotypes identified by nucleotide sequencing analysis and reverse line blot (RLB).

**Results:**

The overall frequency of HPV infection was 36.5% (35 out of 96) in samples negative for intraepithelial lesions or malignancy (NILM), 80% (181 out of 226) in low grade SIL and 93.3% (14 out of 15) in high grade SIL (*P* < 0.001). Thirty-five different genotypes were identified among the 230 HPV-positive cases. The Group 1 oncogenic viruses (HPV16, 18, 31, 33, 35, 39, 45, 51, 52, 56, 58 and 59) were found in 21.9, 46.5, and 86.7% of NILM, low grade SIL and high grade SIL, respectively. The Group 2A, including the probably oncogenic virus HPV68, was found in 1 and 0.8% of NILM and low grade SIL, respectively. The Group 2b possibly oncogenic HPVs (HPV34, 53, 66, 67, 70, 73, 82 and 85) were found in 4.2, 21.7 and 26.7% of NILM, low grade SIL and high grade SIL, respectively. The unclassified viruses (HPV12, 42, 54, 55, 61, 62, 81, 83, 84, 89, 90, 91) were detected in 8.3 and 14.6% of NILM and low grade SIL, respectively, and never in high grade SIL.

**Conclusions:**

Group 1 HPVs were mainly prevalent in high grade SIL and low grade SIL while Group 2B were equally distributed among the two groups. The dominant frequency of unclassified HPVs in low grade SIL and NILM and their rarity in high grade SIL suggests their marginal role in cervical neoplasia of the studied population.

## Background

Human papillomaviruses (HPVs) of the genus alpha represent the most common sexually transmitted viruses infecting mucosal epithelial cells of male and female genital tract [1–3]. Alpha HPVs currently comprise 62 genotypes classified in 15 species on the basis of their phylogenetic similarity [4–6]. The oncogenic risk of mucosal HPVs has been established by large epidemiological studies investigating the different prevalence of specific genotypes in normal cytology, in low and high grade cervical intraepithelial lesions (SIL) and in cervical carcinoma [7–9]. Twelve viruses (HPV16, 18, 31, 33, 35, 39, 45, 51, 52, 56, 58 and 59) have been found significantly associated with cervical carcinoma and classified as Group 1 “carcinogenic to humans”, one virus (HPV68) as Group 2A “probably carcinogenic to humans”, 12 viruses (HPV26, 53, 66, 67, 70, 73, 82, 30, 34, 69, 85, 97) as Group 2B “possibly carcinogenic to humans” and two viruses (HPV6 and 11) as Group 3 “unclassifiable as to carcinogenicity in humans” [10]. Several other HPV genotypes are unclassified regarding to their epidemiologic oncogenic risk although few of them have been shown to bind and to ubiquitinate p53 oncosuppressor with the same efficiency as the Group 1 oncogenic viruses [11, 12].

A meta-analysis of HPV genotype distribution among 115,789 women positive for HPV infection, including normal cytology, atypical squamous cells of undetermined significance (ASCUS), low grade SIL, high grade SIL and invasive cervical cancer cases, showed limited difference in the prevalence of Group 1 HPV type distribution among all groups while HPV16, HPV18 and 45 were relatively high frequent in cervical carcinoma [7]. Moreover, a large meta analysis, comprising above one million women with normal cervical cytology, showed that Group 1 HPV genotypes were found to be the most common viruses in the general female population worldwide, accounting for 70% of HPV infections in normal cytological samples [13]. Some HPV genotypes belonging to the Groups 2A and 2B, namely HPV26, 67, 68, 69, 73 and 82, were found also relatively common in invasive cervical cancer compared to normal cytology [2]. Conversely, Group 2A/2B HPV53 and 66 were found more common in normal cytology and low grade SIL than in invasive cervical cancer. Among viruses with unclassified risk the HPV61, 62, 84 and 89 have been found to be relatively uncommon in normal cytology and invasive cancer and more frequent in low grade SIL [2].

In Italy, several studies evaluating the HPV prevalence and genotype distribution have been performed among women enrolled in organized and in opportunistic screening programs [14–17]. All the studies confirmed the high prevalence of Group 1 HPV genotypes, particularly HP16, 31 and 18 in SIL and invasive cervical cancer [17–19]. However, some unclassified HPVs have been also found to be relatively common in women with normal cytology. In particular, Tornesello et al. observed that several unknown risk viruses were present in low-grade SIL (HPV30, 32), in high-grade SIL (HPV62, 90), and in a small percentage of cervical carcinoma (HPV62) [16, 20]. More recently, Del Prete et al. reported that among 2149 women enrolled in Apulia region the HPV42 was the most common genotype followed by HPV16 with frequency rates of 10.7 and 8.9%, respectively [21].

In the present study we aimed to expand previous analyses on the distribution of alpha HPV genotypes among Italian women referred to the colposcopy outpatient clinic of the Istituto Nazionale Tumori of Napoli after an abnormal cytological diagnosis. We used broad spectrum amplification technology followed by nucleotide sequencing analysis in order to identify known and unknown HPV genotypes infecting the analyzed women population. All HPVs not included in the Group 1, 2A, 2B and 3 have been designed as unclassified HPVs in the present study.

## Methods

### Patients and samples

Cervical cytological samples were collected in liquid-based PreservCyt (Hologic Inc., Marlborough, MA) from 337 Italian women which following a primary abnormal cytological test were referred for a colposcopy examination and directed biopsy at the Istituto Nazionale Tumori “Fond Pascale” from January 2015 to December 2017. All women enrolled in the study self-reported to be not HIV positive or pregnant. Cytology results were recorded according to the Bethesda system as negative for intraepithelial lesions and malignancy (NILM), low grade squamous intraepithelial lesion (SIL) and high grade SIL. Histological diagnoses were available for 224 women and cervical lesions were classified as cervical intraepithelial neoplasia of grade 1, 2 and 3 (CIN1, 2 and 3).

This study was approved by the Institutional Scientific Board of the Istituto Nazionale Tumori “Fond Pascale”, and it is in accordance with the principles of the Declaration of Helsinki. All patients provided written informed consent.

### DNA isolation

PreservCyt specimens were vortexed briefly, divided into two 2-ml aliquots and centrifuged 5 min at 12′000 g. The cell pellet was washed twice with phosphate buffered saline (PBS Buffer, 137 mM NaCl, 2.7 mM KCl, 8 mM Na2HPO4, and 2 mM KH2PO4, pH 7.4) and resuspended in 100 μl of lysis buffer (50 mM Tris-HCl pH 8.5, 1 mM EDTA, 0.5% Tween20) containing proteinase K (200 μg per ml). Cell lysates were digested at 60 °C for 30 min. DNA was purified by phenol and phenol-chloroform-isoamyl alcohol (25:24:1) extraction and concentrated by ethanol precipitation in 0.3 M sodium acetate (pH 4.6). The quantity of isolated DNA was assessed using the spectrophotometer Nanodrop 2000C (Thermo Fisher Scientific, Waltham, MA).

### Broad spectrum HPV amplification and genotyping

Nucleic acid integrity was assessed by PCR amplification of TP53 gene exon 7 which rendered all 337 samples suitable for further analysis [22]. HPV detection was carried out by nested PCR using the MY09/11 consensus primer pairs [23] for the outer reaction (~ 450 bp) and the MGP primer system [24] for the inner reaction (~ 150 bp) [25]. The method has been evaluated 95% proficient for detection of HPV16, 18, 31, 33, 35, 39, 45, 52, 56, 58, 59, 66 and 68b with a specificity above 97% using a proficiency panel of HPV plasmids in the context of the 4th WHO HPV LabNet Proficiency Study for Evaluating HPV DNA Typing Methods (2010), [26]. A negative control sample, made of a reaction mixture without template DNA, was included in every set of five clinical specimens for each PCR run.

The amplified DNA was subjected to electrophoresis on a 7% polyacrylamide gel followed by staining with ethidium bromide and image analysis by the Gel Doc gel imaging system (Bio-Rad Laboratories Inc., Hercules, CA). HPV genotypes were identified by direct automated DNA sequencing analysis of MGP amplified products using both the forward GP5+ and the reverse GP6+ oligoprimers [27] at Eurofins Genomics GmbH (Ebersberg, DE). HPV genotypes were identified by alignment of HPV sequences with those present in the GenBank database using the BLASTn software (http://www.ncbi.nlm.nih.gov/blast/html). The DNA samples showing multiple peaks on the pherograms, compatible with multiple infections, were re-amplified using for the inner reaction biotinylated GP5+/GP6+ primers and resulting amplimers subjected to the reverse line blot assay (Qiagen Manchester Ltd., UK) for the detection of 18 HPV genotypes as described previously [28].

### Statistical analyses

The statistical analysis was performed using the Epi Info 6 Statistical Analysis System Software (Version 6.04b, 1997, Centers for Disease Control and Prevention, USA). Unpaired *t* test was used for comparisons of continuous variables (i.e. age); Yates-corrected χ^2^ test and, where appropriate, two-sided Fisher’s exact test were used for comparison of categorical data. Differences were considered to be statistically significant when *P* values were less than 0.05.

## Results

The study included 337 women with a mean age of 37.6 (± 10.9) years diagnosed with normal cytology (*n* = 96), low grade SIL (*n* = 226) and high grade SIL (*n* = 15). The histological analysis was available for 224 women and rendered 82 NILM, 129 CIN1 and 13 CIN2/3 diagnoses, respectively (Table 1). Overall HPV DNA sequences were detected in 230 out of 337 (68.3%) samples (Table 1). The mean age of HPV-negative and HPV-positive women was 38.5 (±11.8) and 37.2 (±10.6) years, respectively.

**Table 1:** Cytological and histological diagnosis of cervical scrapes and biopsies stratified by HPV status

	Cases n (%)	HPV positive n (%)	HPV negative n (%)
Cytology	337	230 (68.3)	107 (31.8)
NILM*	96 (28.5)	35 (15.2)	61 (57.0)
LSIL*	226 (67.1)	181 (78.7)	45 (42.1)
HSIL*	15 (4.5)	14 (6.1)	1 (0.9)
Histology^a^	224	161 (71.9)	63 (28.1)
NILM*	82 (36.6)	54 (65.8)	28 (34.2)
CIN1*	129 (57.6)	95 (73.6)	34 (26.4)
CIN2/CIN3*	13 (5.8)	12 (92.3)	1 (7.7)

*NILM negative for intraepithelial lesions or malignancy, *LSIL* low grade SIL; *HSIL* high grade SIL; *CIN1, 2 and 3* cervical intraepithelial neoplasia grade 1, 2 or 3

^a^The HPV status of each histological biopsy has been determined on the corresponding cytological sample

Among analyzed samples, 41.3% were positive for Group 1 HPV genotypes, 0.9 and 16.9% for Group 2A and Group 2B, respectively, and 2.7% for Group 3 HPV genotypes. Unclassified viruses were found in 12.2% of samples and represented 17.8% of all infections (Table 2).

**Table 2:** Frequency of group 1, 2A, 2B, 3 and unclassified HPV genotypes among NILM, low grade SIL and high grade SIL

Species	HPV genotype^a^	All samples *n* = 337 (%)	NILM *n* = 96 (%)	LSIL *n* = 226 (%)	HSIL *n* = 15 (%)
	HPV Negative	107 (31.8)	61 (63.5)	45 (20.0)	1 (6.7)
	HPV Positive	230 (68.3)	35 (36.5)	181 (80.0)	14 (93.3)
	Group 1				
A9	HPV16	40 (11.9)	5 (5.2)	30 (13.3)	5 (33.3)
A7	HPV18	13 (3.9)	2 (2.1)	10 (4.4)	1 (6.7)
A9	HPV31	18 (5.3)	0	17 (7.5)	1 (6.7)
A9	HPV33	11 (3.3)	1 (1.0)	9 (3.9)	1 (6.7)
A9	HPV35	2 (0.6)	0	1 (0.4)	1 (6.7)
A7	HPV39	7 (2.1)	1 (1.0)	5 (2.2)	1 (6.7)
A7	HPV45	7 (2.1)	0	7 (3.1)	0
A5	HPV51	6 (1.8)	0	5 (2.2)	1 (6.7)
A9	HPV52	10 (3.0)	4 (4.2)	6 (2.7)	0
A6	HPV56	10 (3.0)	3 (3.1)	5 (2.2)	2 (13.3)
A9	HPV58	8 (2.4)	3 (3.1)	5 (2.2)	0
A7	HPV59	7 (2.1)	2 (2.1)	5 (2.2)	0
	Total Group 1	139 (41.3)	21 (21.9)	105 (46.5)	13 (86.7)
	Group 2A				
A7	HPV68	3 (0.9)	1 (1.0)	2 (0.8)	0
	Group 2B				
A6	HPV53	22 (6.5)	2 (2.1)	19 (8.4)	1 (7.1)
A6	HPV66	21 (6.2)	1 (1.0)	17 (7.5)	3 (21.4)
A9	HPV67	3 (0.9)	0	3 (1.3)	0
A7	HPV70	5 (1.5)	0	5 (2.2)	0
A11	HPV73	2 0.6)	0	2 (0.9)	0
A5	HPV82	2 (0.6)	1 (1.0)	1 (0.4)	0
A11	HPV34	1 (0.3)	0	1 (0.4)	0
A7	HPV85	1 (0.3)	0	1 (0.4)	0
	Total Group 2B	57 (16.9)	4 (4.2)	49 (21.7)	4 (26.7)
	Group 3				
A10	HPV6	8 (2.4)	2 (2.1)	6 (2.7)	0
A10	HPV11	1 (0.3)	0	1 (0.4)	0
	Total Group 3	9 (2.7)	2 (2.1)	7 (3.1)	0
	Unclassified				
A3	HPV81	7 (2.1)	1 (1.0)	6 (2.7)	0
A1	HPV42	5 (1.5)	0	5 (2.2)	0
A3	HPV62	6 (1.8)	1 (1.0)	5 (2.2)	0
A10	HPV55	4 (1.2)	1 (1.0)	3 (1.3)	0
A3	HPV89	4 (1.2)	3 (3.1)	1 (0.4)	0
A8	HPV91	4 (1.2)	0	4 (1.8)	0
A13	HPV54	3 (0.9)	0	3 (1.3)	0
A14	HPV90	3 (0.9)	1 (1.0)	2 (0.9)	0
A3	HPV84	2 (0.6)	1 (1.0)	1 (0.4)	0
A3	HPV61	1 (0.3)	0	1 (0.4)	0
A3	HPV83	1 (0.3)	0	1 (0.4)	0
B1	HPV12	1 (0.3)	0	1 (0.4)	0
	Total Unclassified	41 (12.2)	8 (8.3)	33 (14.6)	0

^a^Type-specific prevalence includes HPVs in single or multiple infections

The most common genotypes were HPV16 (11.9%), HPV31 (5.3%), HPV18 (3.9%), HPV33 (3.3%), HPV52 (3%), HPV56 (3%) HPV58 (2.4%) and HPV59 (2.1%) belonging to Group1; HPV53 (6.5%) and HPV66 (6.2%) of the Group 2; HPV6 (2.4%) of the Group 3 and unclassified HPV type 81 (2.1%). The frequency of all other genotypes was below 2% of all HPV infections (Table 2).

The higher prevalence of HPVs (71.3%) was observed among women in the age group 18–30, *P* = 0.659. Single HPV infections were found in 59.8 and 63.4% of the women aged 18–30 and ≥ 31 years, respectively. Multiple infections were observed in 11.5 and 4.5% of women aged 18–30 and ≥ 31 years, respectively. HPV16 was the most common type found in the two age groups (11.5 and 11.9%, respectively) followed by HPV66 (8.1 and 5.5%), HPV31 (6.9 and 4.5%), HPV 53 (5.8 and 8.4%), HPV58 (5.8% in 18–30 age group), HPV18 (4.6 and 4%) and HPV56 (4.6 and 2.5%).

**Fig. 1:**
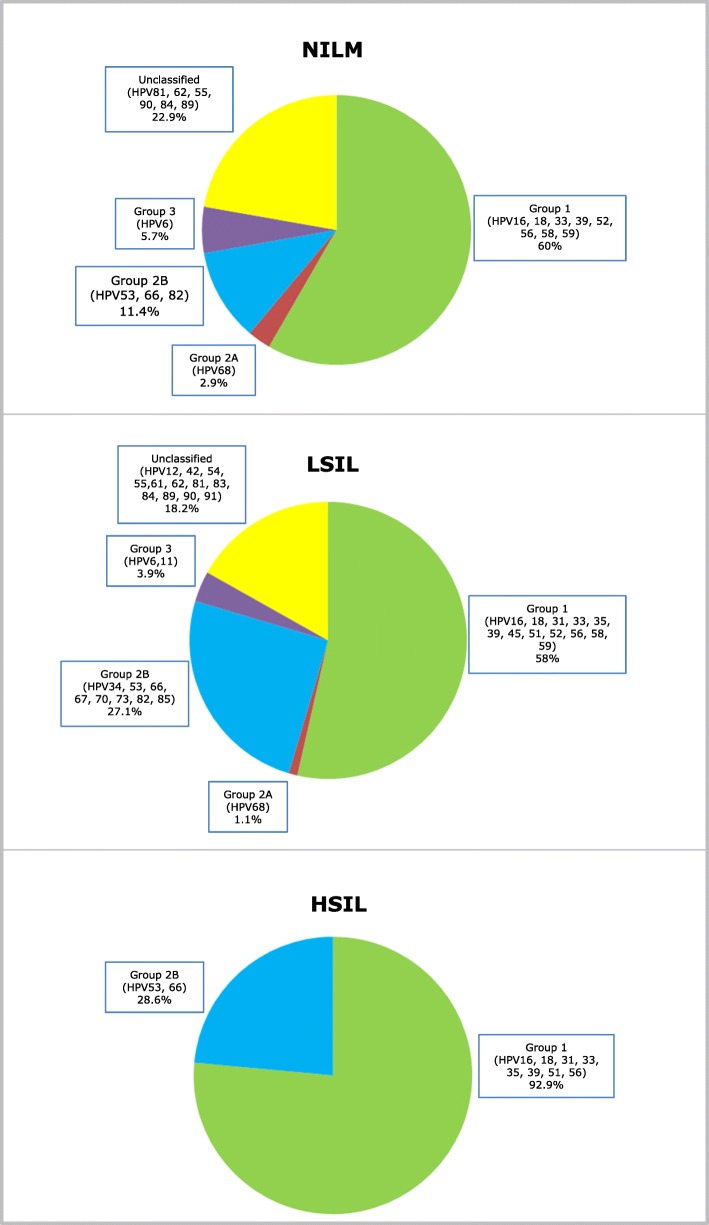
Distribution of Group 1, 2A, 2B, 3 and unclassified HPVs in NILM, low grade SIL (LSIL) and high grade SIL (HSIL). Representativeness of the genotypes contained in each Group is expressed as percentage of all HPV positive cases in each category

According to cervical cytology, the prevalence of HPV infection was 36.5, 80 and 93.3% in patients with NILM, low grade SIL and high grade SIL respectively (Table 2). Group 1 HPV genotypes were detected in 21.9, 46.5, and 86.7% of NILM, low grade SIL and high grade SIL, respectively. Group 2B HPV genotypes were identified in 4.2, 21.7 and 26.7% of NILM, low grade SIL and high grade SIL. Group 3 HPV types were detected in 2.1 and 3.1% of NILM and low grade SIL but in none high grade SIL. Moreover, unclassified HPV types were found in 8.3 and 14.6% of NILM and low grade SIL, respectively, but not in high grade SIL (Table 2). Figure 1 shows the relative frequency of Group 1, 2A and 2B as well as of unclassified HPV genotypes among all HPV-positive NILM, low grade SIL and high grade SIL.

Multiple infections containing at least one HPV genotype of Group 1 were found in 4.5% of cases, while those containing no high risk HPV genotypes represented 1.5% of all analyzed samples. Multiple infections were observed in 2.1% of NILM, 6.6% of low grade SIL and 20% of high grade SIL.

HPV types targeted by the nonavalent HPV vaccine (HPV6, 11, 16, 18, 31, 33, 45, 52 and 58) were detected, alone or in association with other genotypes in 17.7% (17/96), 40.3% (91/226) and 53.3% (8/15) of NILM, low grade SIL and high grade SIL, respectively.

## Discussion

This study provides a comprehensive information on the HPV prevalence and genotype distribution among a cohort of Italian women which were referred to a single Centre for colposcopy following a diagnosis of abnormal cytology. As expected, the overall prevalence of high risk HPVs (Group 1), particularly HPV16, was significantly higher among high grade SIL (86.7%) compared to low grade SIL (46.5%) and NILM (17.7%), *P* < 0.001. Moreover, the probably and possibly carcinogenic HPVs (Group 2A and 2B) were found in 5.2, 22.6 and 26.7% of NILM, low grade SIL and high grade SIL, respectively.

As expected, the infection rates of both high and low risk viruses observed in our study are comparable to that obtained in other studies including Italian women participating in opportunistic screenings but higher than those observed among Italian women attending organized cervical cancer screening programs [17, 19].

Interestingly, we have identified 12 unclassified genotypes in 8.3% of NILM and in 14.6% of low grade SIL but in none of high grade SIL. The inverse correlation between infection frequency and disease severity suggests a limited role for such viruses in cervical carcinogenesis in the analyzed population. Among the unclassified HPVs the genotypes 81, 42, 62 and 91 were the most frequent being found in 2.7, 2.2, 2.2 and 1.8% of low grade SIL, respectively, while the genotype 89 was the most frequent (3.1%) among NILM cases. Despite the low rate of unclassified HPV genotypes in high grade SIL among the women included in the present study, it is important to report that unclassified HPV 54, 61, 62 and 81 represented 27.8% of all infections among HIV-positive Italian women diagnosed with high grade SIL, suggesting that compromised immune system could be not able to limit the “weak oncogenic” activity of some unclassified viruses [29]. Accordingly, Garbuglia et al. found that HPV62 and HPV81 were associated, as single infections, with 9.1 and 4.5% of high grade SIL, respectively, among HIV-positive women [30]. However, the frequency of unclassified HPV genotypes and their oncogenic risk remains underestimated because they are not included in the HPV assays commonly used to detect and characterize HPV genotypes in the majority of studies performed among HIV-negative and HIV-positive women [31, 32].

Cell transformation by HPVs mainly relies on the ability of the viral E6 protein to bind and degrade the p53 oncosuppressor. Mesplède et al. (2012) by performing a quantitative measurement of p53 degradation by the E6 of 29 different HPV genotypes showed that all Group 1 HPVs and several Groups 2A and 2B genotypes (HPV26, 30, 34, 53, 66, 68, 69, 70, 73, 82, 97) were able to suppress p53 by binding and degradation [12]. Studies evaluating the ability of E6 proteins encoded by unclassified HPVs to bind p53 are warranted in order to understand the oncogenic potential of such viruses.

According to our results, the dynamic of unclassified HPVs seems to be stable over the years. In fact, a previous study performed in 2006 in our Centre showed that HPV62 and 81 were the most common unclassified genotypes among SIL samples [16, 20].

In the present study, multiple infections were more frequent in low grade (6.6%) and high grade SIL (20%) than in NILM (2.1%). The majority of such infections (75%) contained at least one HPV genotype of Group 1, while in the remaining 25% various combinations of low risk and unknown risk HPV genotypes were detected. Previous studies showed that multiple HPV infections are not associated to the severity of cervical lesions since they were as common in ICC or HSIL as in LSIL or NILM [33]. However, the coinfections of HPV16 and HPV68 caused a significant increase in the risk of high grade SIL and invasive cervical cancer (OR = 16.5, *P* = 0.0002) compared to that found for HPV16 (OR = 1.9, *P* = 0.003) or HPV68 (OR = 3.5, *P* = 0.38) as single infections, suggesting a synergistic effect between the two viruses [33].

Vaccination against high risk HPVs represents a primary prevention measure for anogenital cancers and squamous intraepithelial lesions caused by those HPV types. The recently licensed nonavalent HPV vaccine targets the seven high-risk HPV genotypes most frequently detected in invasive cervical cancer worldwide (HPV16, 18, 31, 33, 45, 52 and 58) and the low risk HPV genotypes 6 and 11 causing benign genital papillomas [34–36]. Considering the frequency of these nine HPVs among the analyzed women, the use of nonavalent vaccine would be able to prevent more than 50% of HPV infections. In particular, vaccination would prevent 40.3 and 53.3% of low grade SIL and high grade SIL, respectively.

An important limitation of this study was that the self-referred women to the gynecologic center were likely not representative of the general population. Indeed, the HPV prevalence in this cohort was much higher than in the Italian cervical cancer screening population. Moreover, the behavioral risk factors, such as the number of sexual partners and condom use, have not been considered, and the follow up of infected women has not been performed precluding the possibility to evaluate the persistence over the time of unclassified viruses. However, the use of broad spectrum consensus primers able to amplify all 62 mucosotropic HPV genotypes allowed to determine the global distribution of risk classified and unclassified HPVs in the study population.

The application of high-throughput sequencing technology and metagenomic analyses have recently enabled the discovery of many novel HPVs and that the HPV heterogeneity in healthy humans is very complex [37, 38]. It remains to be investigated whether the unclassified virus coinfections have a protecting role, by interfering with the activity of oncogenic viruses or by stimulating the immune cross-reaction, or have a synergistic effect with high risk HPVs facilitating cervical neoplasia development [38].

## Conclusions

Our results show that the majority of low and high grade SIL in women referred for colposcopy at gynecological outpatient clinic of our Center are caused by Group 1 HPVs. The relative high prevalence of unclassified HPVs in low grade SIL and NILM and their absence in high grade SIL suggests that these viruses have a marginal role in cervical neoplasia in the general population. However, the identification of unclassified HPVs in HIV positive women with high grade lesions observed in previous studies, suggests that uncommon and unclassified HPV genotypes need to be characterized in immune compromised patients to allow a correct clinical management.

## Abbreviations

HPV: Human papillomavirus
NILM: Negative for intraepithelial lesion or malignancy
PCR: Polymerase chain reaction
SIL: squamous intraepithelial lesion
